# Modeling and Design of a Rear-Mounted Underwater Projector Using Equivalent Circuits

**DOI:** 10.3390/s20247085

**Published:** 2020-12-10

**Authors:** Jinwook Kim, Yongrae Roh

**Affiliations:** 1 Joint Department of Biomedical Engineering, The University of North Carolina at Chapel Hill and North Carolina State University, Chapel Hill, NC 27599, USA; jinwookk@email.unc.edu; 2 School of Mechanical Engineering, Kyungpook National University, Daegu 41566, Korea

**Keywords:** Tonpilz projector, fixed tail mass, elastomer suspension, equivalent circuit, finite element analysis

## Abstract

Tonpilz is a popular transducer for underwater projector arrays for sonar systems. For low-frequency transmission, a larger axial dimension of the conventional Tonpilz transducer is required. However, a bulky and heavy Tonpilz element is not suitable due to limitations in terms of the space and payload of the array platform. To address this problem, we developed a rear-mounted Tonpilz transducer to generate a sub-fundamental resonance in addition to the common longitudinal resonance. For this purpose, we developed a new equivalent circuit model that can reflect all the effects of the key design parameters of the transducer, such as suspension thickness (stiffness), tail mass thickness, and head mass thickness. The impedance and transmitting voltage response were evaluated as performance factors at both resonance frequencies. The validity of the circuit was verified by comparing the analysis results with those from the finite element analysis of the same transducer. Based on the results, the transducer structure was designed to have comparable transmitting performance at both resonance frequencies by employing relatively high suspension stiffness, light tail mass, and heavy head mass. The novel design can permit the dual-band operation of the transducer so that the transducer can operate as a wideband projector.

## 1. Introduction

Tonpilz is a common mid-range frequency (1–50 kHz) underwater transducer that comprises a stacked piezo-element, a radiation mass at the front end, and a tail mass at the opposite end [1]. Since the Tonpilz transducer is a resonant-type narrowband transducer, the operating frequency is determined by a fundamental longitudinal mode resonance, which is inversely proportional to the axial dimension of the transducer [2]. Hence, a larger transducer size with heavier weight is required for a lower single-mode operating frequency. Low operating frequency is often desired for transmitting waves as it produces reduced losses [3]. To produce low-frequency burst waves with desired acoustic intensity and directivity, several hundreds of Tonpilz transducers are employed as source elements for a large-aperture array projector [4]. However, bulky and heavy Tonpilz transducers are not suitable for the array elements due to the payload limit of the array platform [5]. To alleviate this issue, a small-sized single projector design with a resonance mode within, for example, a low-frequency band is required.

Several multi-mode Tonpilz transducer designs demonstrated that operating frequency bands could be tailored by adding structural resonance modes such as flexural vibration of a head mass [6,7,8,9], multiple head mass resonance modes [1,2], bending mode of a piezo-disk on a head mass [10], and head mass cavity-induced modes [11,12]. Although these designs showed a broader operation bandwidth than single-mode Tonpilz transducers, most of them exploited high-frequency bands, i.e., above the fundamental longitudinal resonance frequency. Thus, multi-mode Tonpilz transducers still require a large axial dimension to cover the low-frequency band below the longitudinal resonance frequency [1,2].

**Figure 1:**
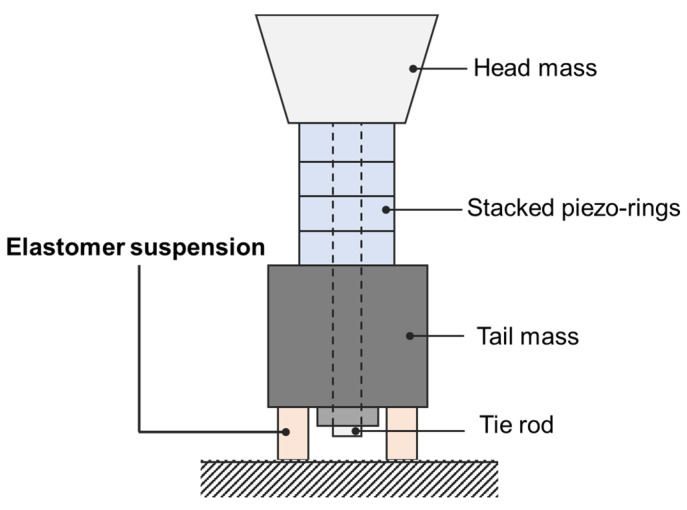
Schematic representation of a rear-mounted Tonpilz projector (4-layer stacked piezo-element as an example). A tail mass is supported by an elastomer suspension to generate a low-frequency resonance below the fundamental longitudinal mode resonance.

In a previous study, we introduced the concept of a fixed tail mass Tonpilz design to mitigate this design problem (Figure 1) [13]. In this design, an elastomer suspension supports a tail mass. Due to the spring constant of a suspension, an additional resonance vibration mode exists in addition to the longitudinal resonance mode of a Tonpilz. This rear-mounted Tonpilz projector generated an elastic fixture-induced whole-body oscillation mode below the longitudinal resonance frequency, which allowed dual-band operation of the transducer [14]. Although the low-frequency mode showed a noticeable lack of transmitting sensitivity (less than −20 dB compared to the longitudinal mode), our preliminary studies, however, showed the feasibility of utilizing such low-frequency resonance for a separate transmission mode. This preliminary study only showed a design concept with the simplified lumped model. Prior to prototyping and experimental validation, further analysis of transmitting characteristics and the effects of key design factors is required.

In this study, we developed a new equivalent circuit (EC) model to reflect all the effects of the key design parameters of the transducer, such as suspension thickness (stiffness), tail mass thickness, and head mass thickness. With the circuit, we analyzed the transmitting performance of the rear-mounted Tonpilz design at both the sub-fundamental and fundamental longitudinal resonance modes. Performance factors were impedance and transmitting voltage response (TVR) spectra. The validity of the circuit was verified by comparing the analysis results with those from the finite element analysis (FEA) of the same transducer. Based on the results, the transducer structure was designed to have comparable transmitting performance at both vibration modes.

## 2. Analysis

A Mason EC model of the rear-mounted Tonpilz structure with a one-dimensional approximation was created for the modeling and analysis, in which the circuit analysis process was coded using MATLAB^®^ (R2019a, Mathworks, Natick, MA, USA). In parallel to the EC model, a FEA was conducted using ANSYS^®^ (Mechanical APDL, ANSYS^®^ Academic Research, Release 19.1, ANSYS, Inc., Canonsburg, PA, USA). Since numerous previous studies have demonstrated that FEA provides a reasonable estimation of Tonpilz transducer performance in the frequency domain, e.g., electrical impedance and transmitting voltage response (TVR) [2,7,8,9,10,11,15,16], FEA results were used as reference data to evaluate the accuracy of the EC models.

### 2.1. Finite Element Model of a Rear-Mounted Tonpilz Projector

**Figure 2:**
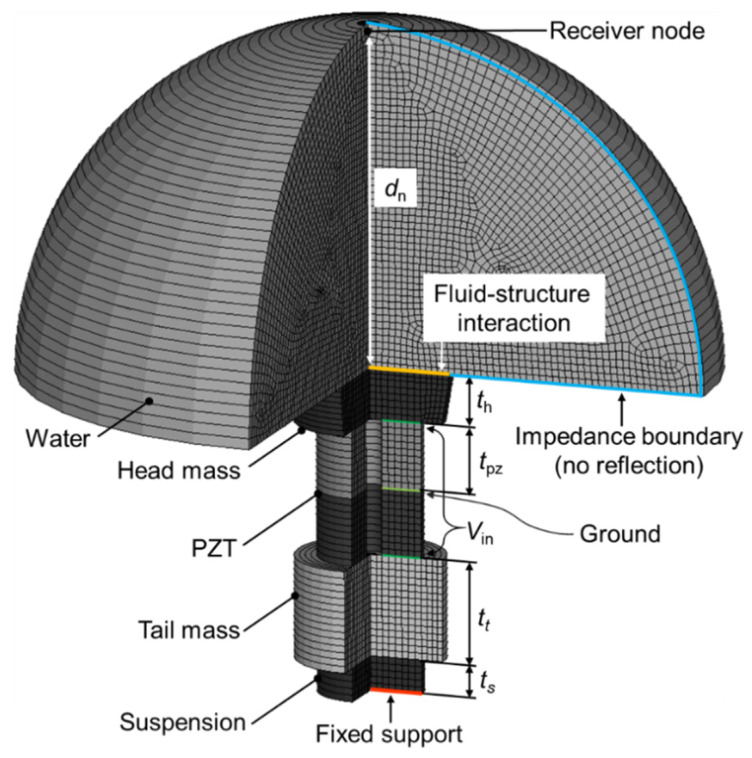
A finite element model (2-D axis-symmetric model) of the rear-mounted Tonpilz transducer.

Figure 2 shows the created two-dimensional (2-D) axis-symmetric model of the transducer and water medium. The basic model has a dimension similar to that used in our preliminary works [13,14]. For simplicity, a tie-rod was removed and only two piezoceramic disks with opposite poling directions were stacked in the model. The averaged element size in the model was kept within 0.03λ, where λ is the wavelength at the longitudinal resonance frequency *f*_L_. The number of elements and nodes were 2023 and 2150, respectively. The head mass diameter was kept at 0.51λ. For harmonic analysis in the normalized frequency range of 0.1*f*_L_–2*f*_L_, 1 V was applied on the electrode layers designated as *V*_in_. The bottom of the suspension layer was fixed by applying a zero-displacement condition on the nodes. The electrical resistance, reactance, and TVR spectra were all calculated. For the calculation of the TVR, a receiver node was set to read complex sound pressures at a distance approximately 1.07λ away in a far-field from the fluid–structure interaction layer. A full sound absorption (no reflection) condition was applied at the outer boundary of the water medium. The material properties and axial dimensions of each component are listed in Table 1 [17].

### 2.2. Distributed Equivalent Circuit Model

**Figure 3:**
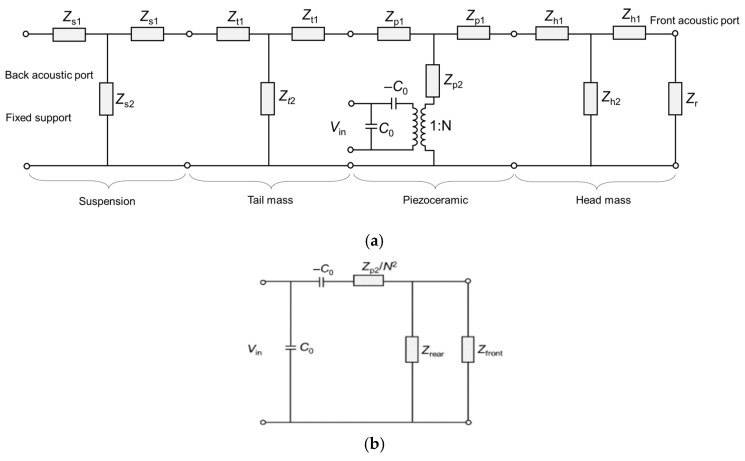
Schematic representation of the equivalent circuit of the rear-mounted Tonpilz transducer. The load elements are defined in Table 2: (**a**) distributed equivalent circuit model; (**b**) rearrangement of the circuit in (**a**) with combined loads. All load impedance elements are referred to as the electrical side.

Based on the Mason EC model of a conventional Tonpilz projector [1], an elastic suspension part was added, which was connected to the tail mass and fixed at the other end. The distributed circuit model is shown in Figure 3a, where the subscripts *r*, *h*, *p*, *t*, and *s* are the radiation load, head mass, piezoceramic, tail mass, and suspension, respectively. The impedance parameters are presented in Table 2. It should be noted that the piezoelectric softening by the negative capacitance (−*C*_0_) was still considered in our model due to the modest aspect ratio (axial dimension/lateral dimension = 1.29) of our stacked piezo-element, which is typically neglected for a long-bar-shaped (aspect ratio > ~5) piezo-element [1,18]. In this case, the most appropriate expression of axial wave speed is given as $1/\sqrt{\rho_{p}s_{33}^{D}}$, which gives a higher wave speed than the case of the long segmented bar due to the relation $s_{33}^{D}<s_{33}^{E}$, where $s_{33}$ is the elastic compliance in the axial direction and *ρ_p_* is the density of the piezoceramic [1]. The superscripts *D* and *E* denote constant electric displacement and constant electric field, respectively. These considerations regarding component dimensions, circuit branch, and associated parameters represent the most important difference from our previous model [14], although a similar rear-mounting concept was applied in the previous EC model [13]. While our previous work introduced a lumped circuit model of the rear-mounted Tonpilz design, taking some errors caused by the simplification to lumped elements [14], this distributed model ensures a more accurate presentation of the transducer components.

The distributed circuit model in Figure 3a can be simplified by repositioning the electrical components on the left side and the mechano-acoustical components on the right side, as shown in Figure 3b. The input impedance and the TVR were calculated over the same frequency range of FEA, i.e., 0.1*f*_L_–2*f*_L_. From Figure 3b, the input admittance (*Y*_in_) can be expressed as shown in Equation (1), and its reciprocal was used to calculate the input electrical impedance (*Z*_in_) [19,20]. To calculate the TVR spectrum, the current through a radiation load, *i*_h_, was used as shown in Equation (2), where the first term is the acoustic power in dB and the second term represents the directivity index (*DI*) of a circular piston source [21]. In Equation (2), *R*_r_ is the radiation resistance of a circular piston, *k* is the wavenumber in water, *a* is the radius of the radiating surface (head mass radius), and *J*_1_ is the first-order Bessel function.

(1) $$Y_{\mathrm{in}}=\frac{1}{Z_{\mathrm{in}}}=i\omega C_{0}+\frac{1}{-\frac{1}{i\omega C_{0}}+\frac{Z_{p2}}{N^{2}}+\frac{Z_{\mathrm{front}}Z_{\mathrm{rear}}}{Z_{\mathrm{front}}+Z_{\mathrm{rear}}}}$$

(2) $$\mathrm{TVR}=10\log\left(\frac{1}{2}{\left|i_{h}\right|}^{2}R_{r}\right)+10\log\left(\frac{{\left(ka\right)}^{2}}{1-J_{1}\left(2ka\right)/\left(ka\right)}\right)+170.8$$

The distributed EC model was used to analyze the variation of the normalized resonance frequency and the TVR of both the sub-fundamental resonance mode and the fundamental longitudinal resonance mode. The suspension thickness, tail mass thickness, and head mass thickness were selected as effective design parameters that affect peak TVR values and their frequencies. During the calculation, one of the selected design parameters was varied from 10% to 200% from the basic model with 20 sub-steps, while the other two parameters were set as 100%, i.e., the basic dimension in Table 1.

## 3. Results

### 3.1. Validation of Equivalent Circuit Models

**Figure 4:**
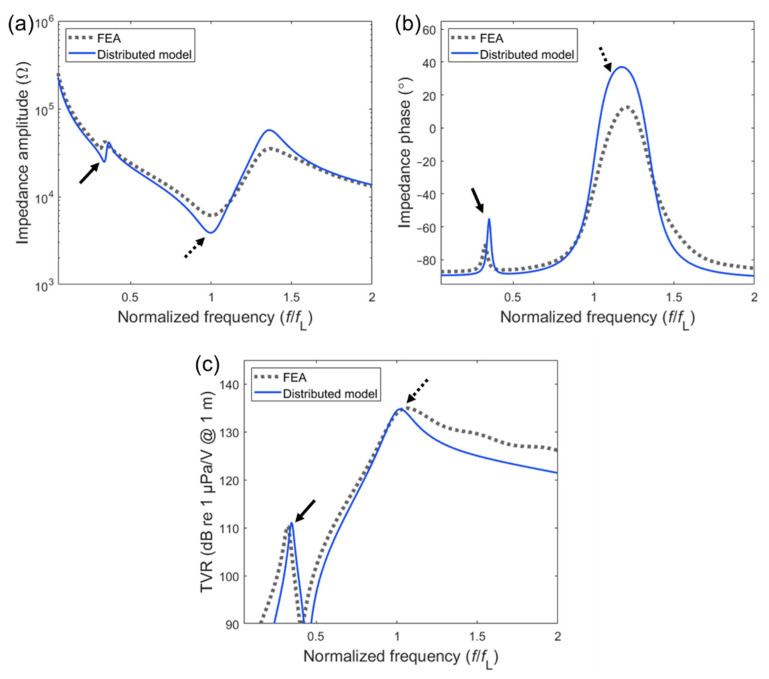
Equivalent circuit and finite element analysis (FEA) simulation of the electrical impedance and the transmitting voltage response (TVR) of the rear-mounted design: (**a**) impedance amplitude spectra; (**b**) impedance phase spectra; (**c**) TVR spectra. The suspension-induced peaks are marked by solid arrows while the fundamental resonance peaks are depicted by dotted arrows.

Since FEA results were used as the reference data, the focus is directed to the difference between the EC analysis data and FEA data. The calculated electrical impedance and TVR spectra are plotted in Figure 4. The electrical impedance is presented as the amplitude in Figure 4a and as the phase angle in Figure 4b over the given frequency range 0.1*f*_L_–2*f*_L_. For a more explicit comparison of the two resonance modes, the frequency was normalized by each longitudinal resonance frequency (*f*_L_) of the FEA and EC analysis, respectively. The values of *f*_L_ from the two analyses showed a discrepancy of less than 3%.

In both the impedance and TVR spectra, distinct peaks were observed, which corresponded to the sub-fundamental and fundamental longitudinal resonance, respectively. In the impedance amplitude spectra, the difference in the sub-fundamental resonance frequencies from the FEA and the EC analysis was less than 3.7%. Such a difference can be attributed to the coupled-mode-induced frequency shift in the FEA because the FEA involved combined 2-D vibrations, whereas the EC considered only pure 1-D vibrations. The impedance amplitude and phase differences were mainly caused by different damping conditions. Such damping conditions include a constant damping ratio of 0.05 for FEA against complex material properties in Table 2 for the EC model. Since these damping conditions were simply adopted from previous works [22], further adjustments may be required for practical fabrication of the transducer. In the TVR spectra in Figure 4c, the level difference between the peaks at 0.32*f*_L_ and those at 1.0*f*_L_ were less than 1 dB.

### 3.2. Suspension Thickness Variation

**Figure 5:**
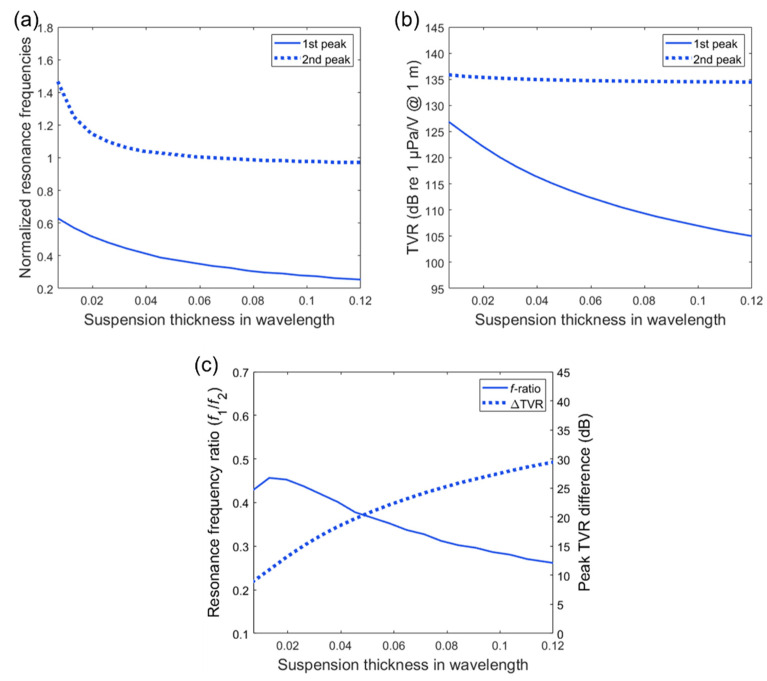
Transmitting characteristics as a function of suspension thickness. The thickness dimension is presented in wavelength and the frequency values are normalized by the fundamental longitudinal resonance frequency *f*_2_: (**a**) normalized resonance frequency variation; (**b**) TVR peak variation; (**c**) the variation in resonance frequency ratio (*f*_1_/*f*_2_) and TVR difference.

The transmitting characteristics at both the sub-fundamental resonance mode (termed “first peak”) and the fundamental longitudinal resonance mode (termed “second peak”) as a function of suspension thickness were analyzed by using the EC model. The suspension thickness was varied from 0.007λ to 0.12λ while the diameter (lateral dimension) was maintained at 0.1λ. As the suspension thickness increases, the normalized frequency of the first peak decreases from 0.63 to 0.25, with a more linear trend than that of the second peak, as shown in Figure 5a. The second peak rapidly decreases from 1.48 to 1.02 when the suspension thickness increases up to 0.052λ. No significant difference exists with the thicker suspension dimensions than this value. This trend correlates with the variation in the stiffness (*k*_s_ = 1/*C*_s_) caused by the change in the suspension thickness because the effective stiffness of the suspension is reciprocal to its thickness ($k_{s}=1/C_{s}=\left(\rho_{s}c_{s}^{2}A_{s}\right)/t_{s}$ from Table 2).

The first peak TVR decreases by more than 20 dB as the suspension thickness increases in the given variation range, as shown in Figure 5b, which is also affected by the stiffness. A higher value of the stiffness generates a higher velocity amplitude of the attached mass (tail mass) in a spring-mass system [21]. Thus, the velocity of the whole Tonpilz oscillation including the head mass increases as well. In comparison with the first peak, the second peak TVR remains close to 135 dB regardless of the changes in the suspension stiffness.

Based on the results in Figure 5a,b, we further investigated the variation trend of the resonance frequency ratio *f*_1_/*f*_2_, where *f*_1_ represents the first peak frequency and *f*_2_ represents the second peak frequency that is equal to *f*_L_ in Section 2 and peak TVR difference (∆TVR) as shown in Figure 5c. The local maximum frequency ratio (*f*_1_/*f*_2_) was obtained as 0.46 when the suspension thickness was 0.013λ. The TVR difference indicates that a shorter suspension thickness (higher stiffness) is essential in reducing the TVR difference of the two resonance modes down to 9 dB.

### 3.3. Tail Mass Thickness Variation

**Figure 6:**
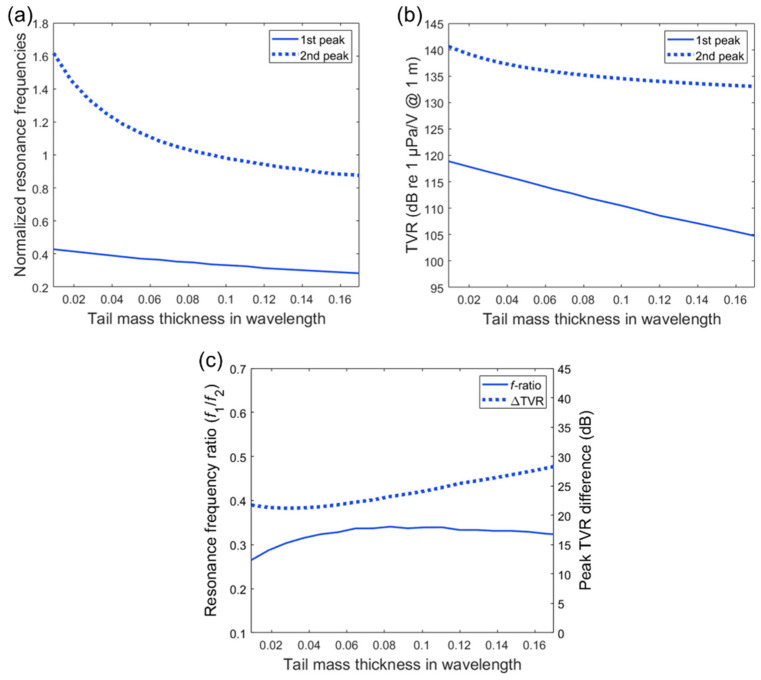
Transmitting characteristics as a function of the tail mass thickness. The thickness dimension is presented in wavelength and the frequency values are normalized by the fundamental longitudinal resonance frequency: (**a**) normalized resonance frequency variation; (**b**) TVR peak variation; (**c**) variation in resonance frequency ratio (*f*_1_/*f*_2_) and TVR difference.

The effects of the tail mass (stainless steel) thickness on the transmitting characteristics were investigated as the thickness was varied in the range of 0.01λ–0.17λ with a constant diameter (0.13λ). Due to the thin thickness in terms of the wavelength, the effect of the tail mass is typically limited to its mass rather than its effective stiffness [1]. Thus, the thickness variation can be considered as a mass variation due to the volume change in the model. The normalized frequency of the second peak varies from 1.6 to 0.88 as a reciprocal function of the tail mass thickness, whereas the normalized frequency of the first peak shows a more linear drop in the smaller range from 0.43 to 0.28 as the tail mass thickness increases, as shown in Figure 6a. With the smallest tail mass, the largest frequency difference of 1.15 was observed, and the largest frequency ratio of 0.34 was observed with the modest tail mass thickness of 0.06, as shown in Figure 6c. The first peak TVR shows a 14 dB difference over the given variation of the tail mass thickness, whereas the second peak shows only a 3 dB difference in Figure 6b. With the tail mass thickness of 0.028λ, as shown in Figure 6c, a local minimum ∆TVR of 21.2 dB was observed. These results indicate that approximately 7 dB of TVR difference between the sub-fundamental and longitudinal resonance mode can be controlled by the tail mass within their frequency ratio range of 0.27–0.34.

### 3.4. Head Mass Thickness Variation

The aluminum head mass thickness was varied from 0.004λ to 0.077λ while the diameter was kept constant as 0.51λ. It should be noted that the distributed circuit model was used without considering the head mass flexural resonance mode since we focused on the TVR peaks and resonance frequency ratio instead of the bandwidth at the higher frequency band (>*f*_2_). The flexural resonance frequency highly depends on the head mass thickness, as shown in the approximated expression in Equation (3), where *c*_h_, *t*_h_, *D*_h_, and *ν*_h_ denote the wave speed, thickness, diameter, and Poisson’s ratio of the head mass, respectively [1]. The mode coupling with a flexural mode typically lowers the longitudinal resonance frequency (*f*_2_) [5,6].

(3) $$f_{\mathrm{flx}}=\frac{1.65c_{h}t_{h}}{D_{h}^{2}\sqrt{1-\nu_{h}^{2}}}$$

**Figure 7:**
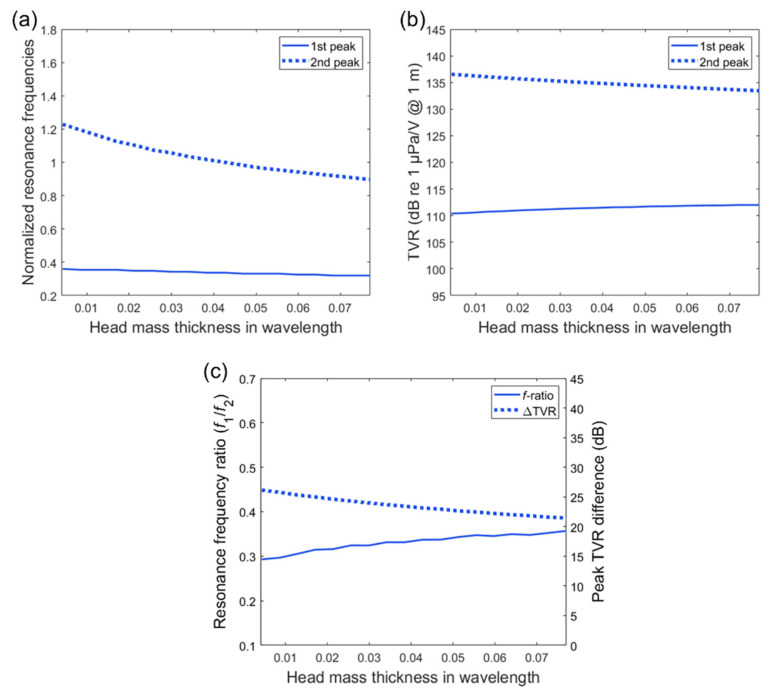
Transmitting characteristics as a function of the head mass thickness. The thickness dimension is presented in wavelength and the frequency values are normalized by the fundamental longitudinal resonance frequency: (**a**) normalized resonance frequency variation; (**b**) TVR peak variation; (**c**) variation in resonance frequency ratio (*f*_1_/*f*_2_) and TVR difference.

As the head mass thickness increases in the given range, the frequency of the first peak decreases by only 11% (0.04), whereas that of the second peak decreases by 27% (0.33), as shown in Figure 7a. The TVR peaks exhibit the opposite trend as the head mass thickness increases. The first peak TVR increases by 1.1 dB, whereas the second peak decreases by 3.1 dB in Figure 7b. In comparison with other components (i.e., suspension thickness and tail mass thickness), the head mass thickness has a relatively weak influence on both the resonance frequency ratio and the TVR difference, as illustrated in Figure 7c. The frequency ratio and TVR difference show a 0.06 increment and 4.7 dB increment, respectively, as the head mass thickness increases in the given range.

## 4. Discussion

**Figure 8:**
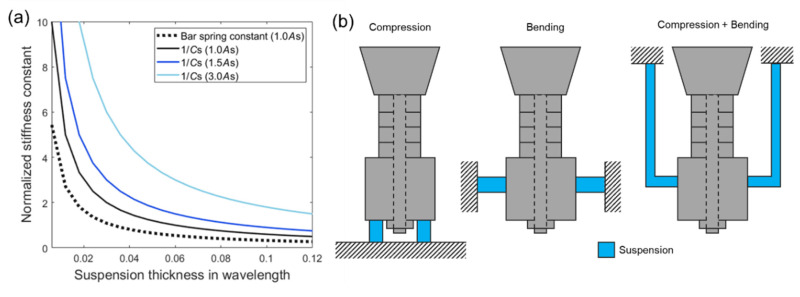
Material and design of a suspension: (**a**) effective stiffness as a function of suspension thickness. The larger cross-sectional areas (1.5*A*_s_ and 3.0*A*_s_) yield higher stiffness; (**b**) suspension design examples (4-layer stacked piezo-element as an example). The same effective stiffness can be designed by adjusting the suspension structure and material.

The suspension thickness determines the effective stiffness, which is a more directly influential parameter affecting the resonance characteristics of the transducer. The thicker suspension has the lower stiffness following the reciprocal relation in Figure 8a. It is worthwhile to note that the effective stiffness was calculated using a motional capacitance *C*_s_, in Table 2, instead of the bar spring constant (*E*_s_*A*_s_/*t*_s_, where *E*_s_, *A*_s_, and *t*_s_ represent Young’s modulus, cross-sectional area, and thickness of the suspension, respectively) due to the comparable axial and lateral dimensions of the suspension disk. There are three controllable parameters to obtain a targeted stiffness: the elastic properties of the material, area, and thickness. In this study, we adopted a synthetic polymer material (a mixture of alumina powder and epoxy bond, longitudinal wave speed of 2700 m/s) whose wave speed could be tailored by changing the alumina powder concentration [23,24,25]. Other synthetic polymers or light metals (e.g., aluminum) can also be used as the suspension material along with a proper dimension adjustment. For example, if an aluminum suspension is used for a similar performance, the suspension is required to be shaped like a thin bar (20% area of the basic design) due to its higher wave speed (6153 m/s). Considering the structure and payload of an array platform, various mounting designs can be used, such as the example designs shown in Figure 8b. We anticipate that the Tonpilz design with a laterally attached suspension, i.e., the second design in Figure 8b, would result in the same acoustic characteristics that we obtained in this study if an appropriate material and dimension are determined for the same effective stiffness.

The main objective in the design of the rear-mounted Tonpilz projector is to maximize the first peak TVR or to minimize ∆TVR. Thus, the order of decreasing importance among the components is from the suspension thickness to the tail mass thickness and, finally, the head mass thickness. For example, the parameters for minimizing TVR difference are summarized in Table 3. To enhance the first peak TVR, the thinner suspension thickness (i.e., higher stiffness) is required, but a cautious control is also needed due to the undesired elevation of both the first and second peak frequencies. From the result of the analysis shown in Figure 5c, the frequency ratio of 0.45 (local maximum) provides a relatively desirable TVR difference of 10.9 dB. Since the high ratio indicates that *f*_2_ is not overly elevated compared to *f*_1_, a suspension stiffness design with the *f*_1_ as ~0.45*f*_2_ is possibly a feasible starting point to achieve the high peak TVR at *f*_1_ while maintaining the targeted operating frequencies. In the rear-mounted Tonpilz design, the control of *f*_1_ is available without a significant axial dimension change, whereas the conventional Tonpilz design requires at least two-fold axial dimension of the rear-mounted design to have a resonance mode in the normalized frequency range of 0.2–0.5.

For designing a tail mass, a thinner mass (lower mass) can be used for higher TVR at *f*_1_. Since the result in Figure 6c shows the local minimum ∆TVR of 21.2 dB with the tail mass thickness of 0.028λ (16.5% percentile in the given range), a low-mass tail can be more desirable, even though it needs cautious control in order to not excessively increase *f*_2_. This trend is the opposite to the prevalent Tonpilz projector design guideline, which states that a large tail mass is desirable as it yields a large head mass velocity for generating more acoustic power [1]. Based on the result of the analysis, the reduced tail mass is more appropriate for the rear-mounted Tonpilz design. This assessment is supported by the results from the head mass variation in Figure 7c as well. By increasing the head mass (reducing a tail-to-head ratio), the first peak TVR becomes closer to the second peak TVR. Moreover, the frequency ratio also shows a beneficial trend (increasing *f*_1_/*f*_2_) for the target performance by increasing the head mass. Thus, for the rear-mounted Tonpilz design with a maximized transmitting sensitivity at the sub-fundamental resonance mode, a relatively lower tail-to-head mass ratio is desirable.

**Figure 9:**
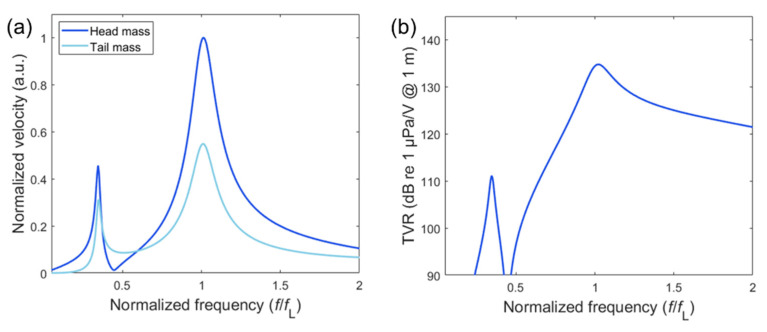
The comparison between the (**a**) velocity spectra (head mass and tail mass) and (**b**) TVR spectrum calculated by the distributed circuit model (basic design).

The velocity spectra of the head and tail mass also show special dynamic characteristics in comparison to the conventional Tonpilz transducers. For the basic model in Figure 2, the tail-to-head velocity ratios at *f*_1_ and *f*_2_ are 1.45 and 1.82, respectively, as shown in Figure 9a. This result is not in agreement with the prevalent relation $\left|u_{h}/u_{t}\right|=M_{t}/M_{h}$ (where *u*_h_ and *u*_t_ represent the velocity of the head and tail masses, respectively) for the conventional Tonpilz design [1], since the tail-to-head mass ratio of our basic model in this study is 4.77. The velocity ratio is much smaller than the mass ratio because of the effect of suspension, and this effect is more dominant at *f*_1_ than *f*_2_. Although the tail-to-head velocity ratio can be an important performance evaluation factor, the evaluation of TVR is essential because of the frequency-dependent radiation characteristic. The head mass velocity at *f*_1_ is 45% of that at *f*_2_, as shown in Figure 9a, whereas the TVR at *f*_1_ is −23 dB of that at *f*_2_, as shown in Figure 9b. This relationship is mainly due to the higher directivity index at a higher frequency, as explained by Equation (2). The overall TVR can be improved by using the multi-layered stacked piezo-elements rather than two layers, as was done in this study, while maintaining the total thickness [26]. For example, the same model with a four-layer piezo-element will generate approximately 6 dB higher TVR in the frequency range of interest compared to the TVR of the model with a two-layer piezo-element. As this constant TVR improvement is independent of frequency and the total thickness is the same, ∆TVR and the frequency ratio will not be strongly affected.

Overall, the EC model developed in this study facilitated our parameter study on the transmitting characteristics of the rear-mounted Tonpilz design. The main difference between our EC analysis and FEA is that the 2-D axis-symmetric finite element model involved a coupled vibration of all components of our basic model (Figure 2) due to the comparable dimensions in the axial and lateral directions. For thin head mass models that possibly apply a noticeable effect of flexural resonance mode near the fundamental mode, EC models with an additional resonance branch or FEA can be used for higher accuracy [5,6]. Despite the higher accuracy of the FEA compared to the EC model, the results obtained in this study imply that the EC model was advantageous over the FEA in terms of the speed and efficiency of the analysis. For this parameter study, the FEA of a 2-D model with 2023 elements took at least 80 times longer than the EC analysis for each case. The overall analysis to cover all the combinations of structural parameter variation will lead to a significant difference in the calculation load, which confirms that the EC model developed in this work can facilitate faster and more efficient analysis and design of the rear-mounted Tonpilz transducer.

## 5. Conclusions

In this study, the transmitting characteristics of a rear-mounted Tonpilz transducer were analyzed by using a distributed EC model. The rear-mounted Tonpilz transducer was characterized by an elastic fixture-induced vibration mode below the longitudinal resonance frequency. The EC model developed herein showed acceptable accuracy and capability of parameter analyses focusing on the variation of resonance frequencies and TVR peaks. Based on the results from the analysis, we conclude that the relatively high-stiffness suspension, light tail mass, and heavy head mass can realize a desirable TVR at the sub-fundamental resonance frequency, i.e., less than 10 dB difference from the TVR at the longitudinal resonance frequency. The new design can allow the dual-band operation of the Tonpilz transducer so that the transducer can work like a wideband projector. Our future work includes prototyping and experimental demonstration of the rear-mounted Tonpilz design.

## Figures and Tables

**Table 1:** Material and structural properties of the finite element model. The parameters, *ρ*, *E*, *υ*, *c*^E^, *e*, and *ε*^S^ denote density, Young’s modulus, Poisson’s ratio, short-circuit elastic stiffness constant, piezoelectric stress constant, and dielectric permittivity at constant strain, respectively.

Component	Suspension	Tail Mass	Piezoceramic	Head Mass
Material	Epoxy composite	Stainless steel	PZT-4	Aluminum
*ρ* (kg/m^3^)	2000	7700	7500	2700
*E* (GPa)	7.93	195	-	69
*υ*	0.38	0.28	-	0.33
$c_{11}^{E},\;c_{13}^{E},\;c_{33}^{E}$ (GPa)	-	-	139, 74.3, 115	-
*e*_31_, *e*_33_ (C/m^2^)	-	-	−5.2, 15.1	-
$\varepsilon_{33}^{S}/\varepsilon_{0}$	-	-	635	-
thickness *t* (λ)	0.065 (*t*_s_)	0.092 (*t*_t_)	0.087 (*t*_pz_)	0.043 (*t*_h_)

**Table 2:** Equivalent circuit parameters. *A*, *n*, and *d*_33_ represent the area, the number of piezoceramic disks, and the piezoelectric strain constant, respectively. *J*_1_ and *H*_1_ are the first-order Bessel function and Struve function, respectively. The function variable *x*_h_ denotes $\left(2\pi f/c_{h\;}\right)r_{h}$, where *r*_h_ is the radius of the head mass.

**Piezoceramic Elements**			
Distributed impedance		$Z_{p1}=i\rho_{p}c_{p}A_{p}\tan\left(nk_{p}t_{p}/2\right)$	
Distributed impedance		$Z_{p2}=-i\rho_{p}c_{p}A_{p}/\sin\left(nk_{p}t_{p}\right)$	
Clamped capacitance		$C_{0}=nA_{p}/t_{p}\varepsilon_{33}^{T}\left(1-k_{33}^{2}\right)$	
Electromechanical coupling coefficient		$k_{33}^{2}=d_{33}^{2}/\left(s_{33}^{E}\varepsilon_{33}^{T}\right)$	
Electromechanical turning ratio		$N=A_{p}/t_{p}d_{33}s_{33}^{E}$	
Wave speed (m/s)		$c_{p}=1/{\left(\rho_{p}s_{33}^{D}\right)}^{\frac{1}{2}}\left(1+i0.013\right)$	
Motional capacitance (C)		$C_{p}=nt_{p}/\left(\rho_{p}c_{p}^{2}A_{p}\right)$	
Mass (kg)		$M_{p}=n\rho_{p}t_{p}A_{p}$	
	**Suspension**	**Tail Mass**	**Head Mass**
Mechanical impedance	$Z_{s0}=\rho_{s}c_{s}A_{s}$	$Z_{t0}=\rho_{t}c_{t}A_{t}$	$Z_{h0}=\rho_{h}c_{h}A_{h}$
Distributed impedance	$Z_{s1}=iZ_{s0}\tan\left(k_{t}t_{t}/2\right)$	$Z_{t1}=iZ_{t0}\tan\left(k_{t}t_{t}/2\right)$	$Z_{h1}=iZ_{h0}\tan\left(k_{h}t_{h}/2\right)$
Distributed impedance	$Z_{s2}=-iZ_{s0}/\sin\left(k_{s}t_{s}\right)$	$Z_{t2}=-iZ_{t0}/\sin\left(k_{t}t_{t}\right)$	$Z_{h2}=-iZ_{h0}/\sin\left(k_{h}t_{h}\right)$
Wave speed (m/s)	$c_{s}=2700\left(1+i0.03\right)$	$c_{t}=5690\left(1+i0.013\right)$	$c_{h}=6153\left(1+i0.013\right)$
Motional capacitance (C)	$C_{s}=t_{s}/\left(\rho_{s}c_{s}^{2}A_{s}\right)$	-	-
Mass (kg)	$M_{s}=\rho_{s}t_{s}A_{s}$	$M_{t}=\rho_{t}t_{t}A_{t}$	$M_{h}=\rho_{h}t_{h}A_{h}$
Radiation impedance	-	-	$Z_{r}=R_{r}+iX_{r}$
Radiation resistance	-	-	$R_{r}=Z_{h0}\left(1-\frac{2J_{1}\left(x_{h}\right)}{x_{h}}\right)$
Radiation mass	-	-	$X_{r}=\frac{2H_{1}\left(x_{h}\right)}{x_{h}}$
	**Combined Circuit Elements**		
Equivalent impedance	$Z_{\mathrm{front}}=\frac{1}{N^{2}}\left[Z_{p1}+Z_{h1}+\frac{Z_{h2}\left(Z_{r}+Z_{h1}\right)}{Z_{h2}+\left(Z_{r}+Z_{h1}\right)}\right]$		
Equivalent impedance	$Z_{\mathrm{rear}}=\frac{1}{N^{2}}\left[Z_{p1}+Z_{t1}+\frac{Z_{t2}\left(Z_{t1}+Z_{s1}+Z_{s2}\right)}{Z_{t2}+\left(Z_{t1}+Z_{s1}+Z_{s2}\right)}\right]$		

**Table 3:** Summary of the parameters for minimizing the peak TVR difference between *f*_1_ and *f*_2_.

Component	Suspension	Tail Mass	Head Mass
Thickness (the calculated range)	0.007 (0.007–0.12)	0.028 (0.01–0.17)	0.077 (0.004–0.077)
*f*_1_/*f*_2_	0.44	0.3	0.36
∆TVR (dB)	9.0	21.2	21.5
